# Acknowledgement to Reviewers of *Bioenginering* in 2015

**DOI:** 10.3390/bioengineering3010008

**Published:** 2016-01-29

**Authors:** Bioengineering Editorial Office

**Affiliations:** MDPI AG, Klybeckstrasse 64, CH-4057 Basel, Switzerland; bioengineering@mdpi.com

The editors of *Bioengineering* would like to express their sincere gratitude to the following reviewers for assessing manuscripts in 2015.

We greatly appreciate the contribution of expert reviewers, which is crucial to the journal’s editorial decision-making process. Several steps have been taken in 2015 to thank and acknowledge reviewers. Good, timely reviews are rewarded with a discount off their next MDPI publication. By creating an account on the submission system, reviewers can access details of their past reviews, see the comments of other reviewers, and download a letter of acknowledgement for their records. In addition, MDPI has launched a collaboration with Publons, a website that seeks to publicly acknowledge reviewers on a per journal basis. This is all done, of course, within the constraints of reviewer confidentiality. Feedback from reviewers shows that most see their task as a voluntary and mostly unseen work in service to the scientific community. We are grateful to our reviewers for the contribution they make.

Aarts, Ronald M.
Baigude, Huricha
Bazylinski, Dennis A.
Branchini, Alessio
Carothers, James
Casañ-Pastor, N.
Ching, Congo Tak Shing
Cinelli, Patrizia
Costa, Rui
De Vrieze, Jo
De-Juan-Pardo, Elena M.
Dijkstra, Paul
Doeven, Egan
Esteves, Sandra
Feng, Yu
Hanai, Taizo
Hasegawa, Shinya
Hastings, Astley
Heo, Min-Suk
Horstkorte, Rüdiger
Huang, Po-Whei
Jayant, Rahul Dev
Jiang, Ning
Jing, Fuyuan
Kao, Katy
Kaviratna, Anubhav
Kovacs, Kornél
Lan, Ethan
Lazzara, Matthew
Leporatti, Stefano
Lewis, Nathan
Li, Guanglin
Li, Hang
Lienen, Tobias
Liu, Jing
Lu, Ting
Matsumoto, Takeshi
Morabito, Francesco Carlo
Nalayanda, Divya
Naseer, Noman
Nuhn, Lutz
O'Connor, José-Enrique
Orellana, Guillermo
Pantaleo, A.M.
Pauss, Andre
Poertner, Ralf
Polacheck, William
Polizzi, Karen
Pusapati, Ganesh
Ragunathan, Kaushik
Ramos, Jose
Ramos, Victoria
Richardson, James W.
Rodriguez, Gabriel
Ruiz-Rodríguez, Juan C.
Saady, Noori M. Cata
Sahm, Kerstin
Samba, Ramona
Schuman, Meredith
Shah, Pratikkumar
Shimizu, Hiroshi
Shirure, Venktesh
Smith, Kerry
Stams, Alfons J. M.
Stratakis, Emmanuel
Svensson, Bo
Tanaka, Katsunori
Tashiro, Yohei
Thomsen, Sune Tjalfe
Wagner, Andreas Otto
Wang, Yang
Wiedemann, Philipp
Wujcik, Evan
Yarema, Kevin
Yoon, Seongkyu
Zengler, Karsten
Zhang, Yalei
Zhang, Ge
Zinn, Manfred

